# Spatial distribution of *Taenia solium* exposure in humans and pigs in the Central Highlands of Vietnam

**DOI:** 10.1371/journal.pntd.0006810

**Published:** 2018-09-20

**Authors:** Dinh Ng-Nguyen, Rebecca Justine Traub, Van-Anh Thi Nguyen, Kathleen Breen, Mark Anthony Stevenson

**Affiliations:** 1 Faculty of Veterinary and Agricultural Sciences, University of Melbourne, Parkville, Victoria, Australia; 2 Faculty of Animal Sciences and Veterinary Medicine, Tay Nguyen University, Dak Lak, Vietnam; 3 Department of Livestock, Montana Veterinary Diagnostic Lab, Bozeman, Montana, United States of America; University of Queensland School of Veterinary Science, AUSTRALIA

## Abstract

**Background:**

*Taenia solium*, a pork-borne parasitic zoonosis, is the cause of taeniasis and cysticercosis in humans. In Vietnam, poor sanitation, the practice of outdoor defecation and consumption of raw/undercooked pork have been associated with infection/exposure to *T*. *solium* in both humans and pigs. The broad-scale geographic distribution of the prevalence of *T*. *solium* varies throughout the country with infection restricted to isolated *foci* in the north and a more sporadic geographic distribution in the Central Highlands and the south. While cross-sectional studies have allowed the broad-scale geographic distribution of *T*. *solium* to be described, details of the geographic distribution of *T*. *solium* at finer spatial scales have not been described in detail. This study provides a descriptive spatial analysis of *T*. *solium* exposure in humans and pigs and *T*. *solium* taeniasis in humans within individual households in village communities of Dak Lak in the Central Highlands of Vietnam.

**Methodology/Principal findings:**

We used Ripley’s K-function to describe spatial dependence in *T*. *solium* exposure positive and negative human and pig households and *T*. *solium* taeniasis exposure positive and negative households in villages within the districts of Buon Don, Krong Nang and M’Drak of Dak Lak province in the Central Highlands of Vietnam. The prevalence of exposure to *T*. *solium* in pigs in Dak Lak province was 9 (95% CI 5 to 17) cases per 1000 pigs at risk. The prevalence of exposure to the parasite in humans was somewhat higher at 5 (95% CI 3 to 8) cases per 100 individuals at risk. Spatial aggregations of *T*. *solium* exposure-positive pig and human households occurred in some, but not all of the villages in the three study districts. Human exposure-positive households were found to be aggregated within a distance of 200 to 300 m in villages in Krong Nang district compared with distances of up to 1500 m for pig exposure-positive households in villages in M’Drak district. Although this study demonstrated the aggregation of households in which either *T*. *solium* exposure- or taeniasis-positive individuals were present, we were unable to identify an association between the two due to the very low number of *T*. *solium* taeniasis-positive households.

**Conclusions:**

Spatial aggregations of *T*. *solium* exposure-positive pig and human households occurred in some, but not all of the villages in the three study districts. We were unable to definitively identify reasons for these findings but speculate that they were due to a combination of demographic, anthropological and micro-environmental factors. To more definitively identify characteristics that increase cysticercosis risk we propose that cross-sectional studies similar in design to that described in this paper should be applied in other provinces of Vietnam.

## Author summary

*Taenia solium* is a pork-bone zoonotic parasite. Humans acquire taeniasis from consumption of raw/undercooked pork contaminated with *T*. *solium* cysticerci. Pigs and humans acquire cysticercosis following consumption of food contaminated with eggs shed from the feces of humans with *T*. *solium* taeniasis. In Vietnam, the geographic distribution of *T*. *solium* varies throughout the country with hotspots or *foci* of infection in communities in the North and a more sporadic distribution in the Central Highlands and the South. While information on the distribution at the regional and provincial level is available, there is no available information on the spatial distribution of *T*. *solium* at fine spatial scales and factors influencing its distribution at fine spatial scales have not been described in detail. In this cross-sectional study, we collected information on the geographic coordinates of study households and utilized spatial analytical techniques to quantify both the fine scale spatial pattern of exposure to *T*. *solium* as well as the tendency for *T*. *solium* exposure-positive households to be located close to other *T*. *solium* exposure-positive households (spatial autocorrelation) in three districts in Dak Lak province. We found that in some of the study villages *T*. *solium* exposure-positive households were more likely to be surrounded by other *T*. *solium* exposure-positive households. Human exposure-positive households were found to be aggregated within a distance of 200 to 300 m in villages in Krong Nang district; whilst spatial aggregation of pig exposure-positive households was found up to distances of 1500 m in villages in M’Drak district. Although households that had either *T*. *solium* exposure- or taeniasis-positive cases were aggregated, we were unable to quantify their spatial association due to the extremely low number of *T*. *solium* taeniasis-positive households. This study shows that in the Central Highlands of Vietnam, *T*. *solium* exposure tend to cluster within *foci*. This information can be used to inform community intervention programs to lower its incidence in both humans and pigs.

## Introduction

*Taenia solium* is a pork-borne zoonosis of major public health and economic importance. The parasite causes cysticercosis/neurocysticercosis in humans and pigs in many low-income communities in Latin America, Africa and Asia [1]. Poor sanitation, allowing pigs to free roam, the practice of outdoor defecation and consumption of raw/undercooked pork are risk factors for *T*. *solium* infection. Cysticercosis infection in humans and pigs occurs due to the accidental ingestion of *T*. *solium* eggs shed through the feces of humans with *T*. *solium* taeniasis (tapeworm carriers). Taeniasis occurs when humans consume raw/undercooked pork with *T*. *solium* cysticerci.

*T*. *solium* infection results in not only an economic burden in low-income communities due to loss of productivity in affected individuals and the cost of treatment, but also losses arising from the condemnation of pig carcasses destined for human consumption. It was estimated that approximately US $185 million and 2.1 million disability-adjusted life years (DALYs) were lost in north India due to human cysticercosis in 2011 [2]. It was estimated that the number of DALYs for human cysticercosis in Mozambique in 2007 [3] was 6.0 per thousand person-years, 0.2 in 2011 in Mexico [4] and 0.7 in 2012 in Tanzania [5]. The pork industry in four provinces of Laos estimated losses of between US $55,000 to 96,000 arising from 20% of carcasses identified as cysticerci-positive over a 21 month period [6]. In Latin America, the number of neurocysticercosis infections has been estimated to be between 11 and 29 million with 1.3 million individuals suffering from neurocysticercosis-related epilepsy [7]. Globally, cysticercosis was estimated to be the cause of over 28,000 deaths in 2010 [8].

Although risk factors for *T*. *solium* taeniasis and cysticercosis include outdoor defecation, the consumption of raw/undercooked pork and allowing pigs to free roam, transmission patterns and the prevalence of the parasite can vary considerably and may prove inconsistent within and/or between regions and communities [9] thus impeding efforts to achieve parasite eradication. Because of the negative impact of *T*. *solium* on human health and the economy [10], controlling the disease is a priority. Understanding the spatial distribution of *T*. *solium* is one important step towards development of effective control strategies. Studies in communities of Latin America and Africa, where *T*. *solium* infection is hyperendemic, identified clustering of *T*. *solium* cysticercosis at both the household and community level [11–13] and a strong geographical association between *T*. *solium* carriers and cysticercosis in humans and pigs [14,15]. Spatial analyses were used to define an appropriate radius for a control area in a community where *T*. *solium* was hyperendemic in Peru. Targeting interventions within this control area was effective in reducing the number of *T*. *solium* carriers and the sero-incidence of porcine cysticercosis [16].

*T*. *solium* is endemic in Vietnam. A systematic review of cross-sectional studies carried out in Vietnam between 1999 to 2011 showed that the prevalence ranged from 0 to 130 *T*. *solium* positive individuals per 1000 individuals at risk [17]. The distribution of *T*. *solium* in Vietnam is characterized by hotspots or *foci* of infection in communities in the northern provinces, including Phu Tho and Bac Ninh [18,19]. In the Central Highlands and the south of the country the distribution of *T*. *solium* is sporadic [20]. While previous studies [15,16,17] have provided useful information at the regional and provincial level, we are aware of no investigations that have investigated the distribution of *T*. *solium* at finer spatial scales. With this background, the aim of this study was to describe the spatial distribution of households in which one or more individual humans or pigs were *T*. *solium* exposure positive and households in which one or more individual (humans) were *T*. *solium* taeniasis positive. Quantitative estimates of the prevalence and geographic distribution of *T*. *solium* exposure and *T*. *solium* taeniasis positive households will provide evidence to allow public health authorities to decide between treatment programs applied at the whole community level as opposed to treatment programs applied at either the individual household or small area level.

## Materials and methods

### Ethics statement

This study was reviewed and approved by the Behavioral and Social Sciences Human Ethics Sub-committee, the University of Melbourne (reference number 1443512) and the Animal Ethics and Scientific Committee, Tay Nguyen University (reference number 50.KCNTY). This study was conducted under the supervision of the local Center for Public Health and the local Center for Animal Health, Dak Lak, Vietnam. This research on pigs was based on the International Guiding Principle for Biomedical Research Involving Animals issued by the Council for the International Organization of Medical Sciences.

### Field procedures

**Fig 1:**
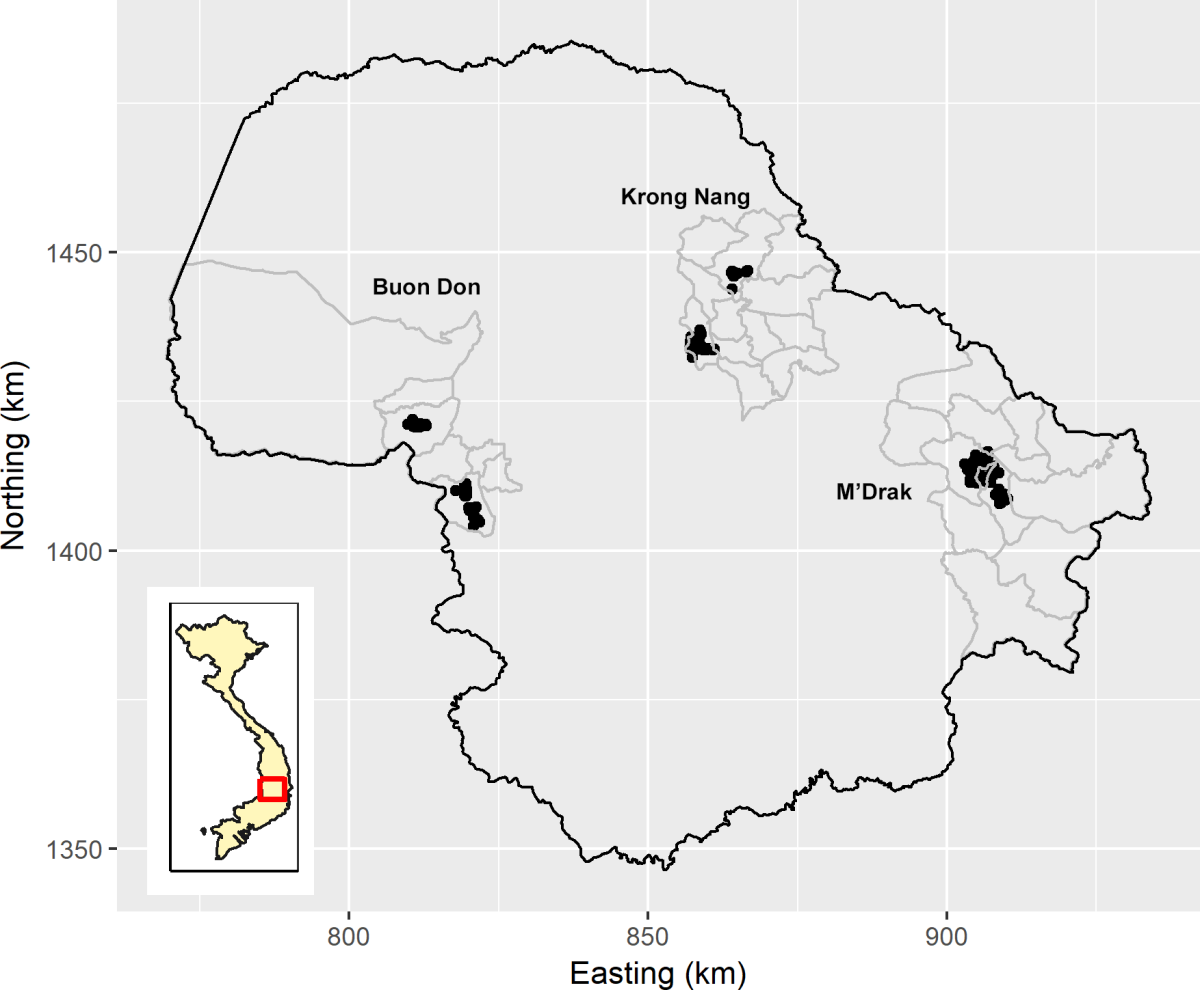
Map of Dak Lak province showing the location of study sites. The black shaded areas show the study locations in Buon Don, Krong Nang and M’Drak districts. This figure was adapted from Ng-Nguyen et al. (2017) [22].

**Fig 2:**
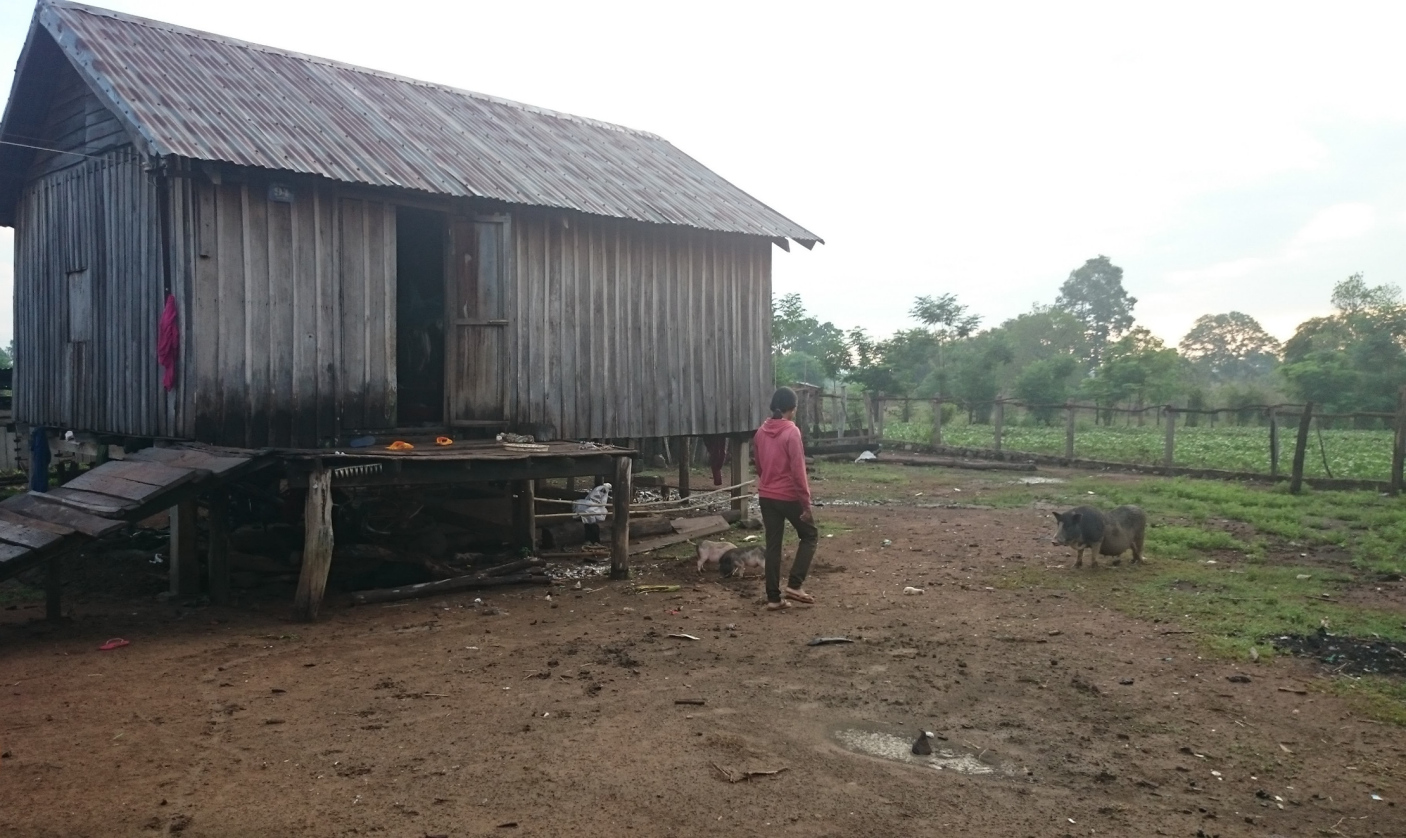
A picture showing a typical household in rural areas in Dak Lak province. The space under the floor of the stilt house is used for the shelter of domestic animals.

The cross-sectional study was carried out between May and October 2015 in Dak Lak province in the Central Highlands of Vietnam. Dak Lak is comprised of 15 districts with approximately 70% of the total population of 1.8 million people living in rural areas [21]. Within the province, three districts namely Buon Don, Krong Nang and M’Drak were chosen as the study sites based on their diverse geographic characteristics (Fig 1). The characteristics of these districts have been described in detail elsewhere [22]. A sampling frame listing the name of all villages in Dak Lak province was obtained from Sub-Department of Animal Health office within the Ministry of Agriculture and Rural Development. Villages eligible for sampling comprised those with more than 1000 pigs, as recorded by the Sub-Department of Animal Health. All eligible villages within each district were assigned a number and two numbers chosen at random to select villages from each district for inclusion in the study. A list of householder names within each selected village was obtained from each village head person, and householder names were assigned a numeric code. A sheet of paper listing numeric household codes for each village were cut into pieces and placed face-down on a table. The village head person was asked to select 50 households at random for human sampling and between 100 and 140 households for pig sampling. All households selected for human sampling and pig sampling were visited several days before the proposed sampling date to obtain consent from householders to take part in the study. Householders eligible for inclusion in the study were individuals who were healthy, not pregnant and over seven years of age. Householders requested to take part in the study signed a consent form. Those that were under 18 years of age were required to provide written consent as well as written consent from either their parents or legal guardians. At the time of consent each study participant was given a labeled stool container, with instructions that the container would be collected on the date of sampling, several days later. Sampling of households (Fig 2) was carried out in two stages. Humans from each of the 50 consenting study households were sampled between May and October 2015; pigs from each of the 100 to 150 consenting study households were sampled between June and October 2015. At the time of each household visit, a questionnaire was administered to each of the study participants soliciting details regarding demography, sanitation and hygiene status, food culture and religion, practice of pig management and the longitude and latitude coordinates of the main doorway of entry of the dwelling used for sleeping.

On the date of sampling, consenting householders were visited by staff from the Sub-Department of Health and 5 mL of venous blood collected into plain clotting tubes from consenting study participants. Stool samples were fixed in 5% potassium dichromate (w/v) for molecular analysis.

Pigs that were pregnant, ill or aged less than 2 months of age were excluded from sampling. Approximately, 10 mL of blood was obtained from the cranial vena cava of each pig into plain blood collection tubes. Blood samples were allowed to clot at ambient temperature prior to centrifugation at 3200 × g for 5 minutes to collect serum. Serum was dispensed into 1.5 mL aliquots and stored at -20°C until analysis.

The longitude and latitude of the main doorway of entry of sampled households was recorded using a handheld global positioning system (GPS) device (Garmin GPSMAP64, Taiwan).

### *Taenia solium* carrier identification

Human stool samples were tested to determine *T*. *solium* tapeworm carriers using a real-time PCR (T3qPCR) described by Ng-Nguyen et al. (2017) [22]. The assay has been reported to have a diagnostic sensitivity of 94% and a diagnostic specificity of 98%.

### Human *T*. *solium* exposure identification

Human serum samples were tested for the presence of antibody against *T*. *solium* cysticerci using a lentil-lectin purified glycoprotein-enzyme-linked immunoelectrotransfer blot (LLGP-EITB). This assay has a diagnostic sensitivity of 98% and a diagnostic specificity of 100%. The LLGP-EITB was performed using the methodology described by Tsang et al. (1989) [23].

### Pig *T*. *solium* exposure identification

Pig serum samples were tested for the presence antibody against *T*. *solium* cysticerci using rT24H antigen in the enzyme-linked immunoelectrotransfer blot (EITB) format. The rT24H-EITB assay showed no cross-reaction to *T*. *hydatigena* and had a diagnostic sensitivity and specificity of 100% when tested on 29 cysticercosis-negative USA pig sera, 12 necropsy-positive *T*. *solium*-positive Peruvian pig sera and four *T*. *hydatigena* necropsy-positive Vietnamese pig sera [24]. The performance of the rT24H-EITB assay was carried out using the methodology described by Noh et al. 2014 [25]. Positive samples resulting from the rT24H-EITB assay were confirmed using the LLGP-EITB assay.

### Spatial data analyses

Details collected during each of the household visits and laboratory test results were stored in a relational database (Microsoft Access 2007, Microsoft Corporation, Redmond, USA). Longitude and latitude coordinates of household locations (recorded in degrees, minutes and seconds) were converted to decimal degrees and re-projected to the Universal Transverse Mercator Zone 48N projection using the World Geodetic System 1984 datum.

Analyses were carried out to describe the spatial characteristics of: (1) *T*. *solium* exposure-positive and *T*. *solium* exposure-negative households for humans; (2) *T*. *solium* exposure-positive and *T*. *solium* exposure-negative households for pigs; and (3) human or pig *T*. *solium* or *T*. *solium* taeniasis-positive and negative households.

Our classification of *T*. *solium* exposure-positive households for humans and pigs was based on the LLGP-EITB and rT24H-EITB assays, respectively. Classification of *Taenia*-positive households was based on parallel interpretation of the test results of the LLGP-EITB, rT24H-EITB and T3qPCR assays (positive for either *T*. *solium* exposure or *T*. *solium* taeniasis).

Ripley’s K-function [26] provides a summary measure of spatial dependence among point locations as a function of their Euclidean distance. The K-function is defined as the expected number of points that are located within a distance *h* of an arbitrarily selected point location, divided by the overall density of points [27]. Where there is spatial dependence in a point pattern, point events are likely to be surrounded by other point events and, for small vales of distance *h*, *K*(*h*) will be relatively large. Conversely, if point events are regularly spaced, each point is likely to be surrounded by empty space and, for small values of distance *h*, *K*(*h*) will be small. To facilitate inference, we developed separate K-function plots for *T*. *solium* exposure-positive and *T*. *solium* exposure-negative households. For each value of *h* we then calculated the K-function difference as *D*(*h*) = *K*(*h*)_*positive*_ – *K*(*h*)_*negative*_. If exposure-positive households were spatially aggregated, over and above that of the exposure-negative households, then *D*(*h*) will appear graphically as peaks (or troughs) as a function of distance.

Three sets of K-function analyses were carried out for: (1) human *T*. *solium* exposure-positive households and human *T*. *solium* exposure-negative households; (2) pig *T*. *solium* exposure-positive households and pig *T*. *solium* exposure-negative households; and (3) *T*. *solium* taeniasis exposure positive and *T*. *solium* taeniasis negative households.

Monte Carlo simulation was used to construct critical envelopes for each K-function difference plot. Here, we randomly assigned the observed number of positive households across the population of study household locations and re-computed *D*(*h*) each time. The critical envelopes are based on 1000 Monte Carlo simulations of the data. Departures of the observed value of *D*(*h*) above the limits of the upper and lower critical envelopes provided an indication of spatial aggregation of exposure-positive households beyond that which would be expected by chance, and at what spatial scale.

## Results

### General data description

The total numbers of households visited for collecting samples from humans and pigs, respectively, were 190 and 408 in Buon Don, Krong Nang and M’Drak districts. Within the 190 households, a total of 342 individuals consented to participate in the study. The number of pigs sampled from the 408 households was 1281. Four of the 190 households (2.1%, 95% CI 0.6 to 5.6) housed *T*. *solium* tapeworm carriers and the percentages of households housing individuals and pigs that were *T*. *solium* exposure positive was 8.9% (17/190, 95% CI 5.5 to 14) and 2.7% (11/408, 95% CI 1.4 to 4.9), respectively. Amongst the 11 *T*. *solium* exposure-positive households for pigs, there was one household that had more than one pig antibody-positive to *T*. *solium* cysticerci; all other exposure-positive households had a single pig that was seropositive. All *T*. *solium* exposure-positive households for humans had a single individual that was antibody-positive. Of the 561 households that were visited for either human or pig sampling, 31 had either humans or pigs that were either human *T*. *solium* exposure-positive, pig *T*. *solium* exposure-positive or *T*. *solium* taeniasis positive (Table 1). There were 29 households with single infections; one household with exposure-positive pigs and one household had an individual infected with *T*. *solium* taeniasis and an individual that was *T*. *solium* exposure-positive. Human *T*. *solium* exposure- and taeniasis-positive households were present in all three districts. There was no pig *T*. *solium* exposure-positive households in Buon Don.

**Table 1:** Spatial characteristics of *T*. *solium* exposure and taeniasis. Data structure of household and individual sampling.

Infection status	Humans	Pigs	Total
Total number of households visited	190	408	561 ^a^
*T*. *solium* exposure-positive households (pigs)	-	11	11
*T*. *solium* exposure-positive households (humans)	17	-	17
Taeniasis-positive households	4	-	4
*Taenia*-positive households	-	-	31 ^b^
Total number of tested study subjects	342	1281	1623
Total *T*. *solium* exposure-positive subjects	17	12	29
Total taeniasis-positive subjects	4	-	4

^a^ Total of visited households for sampling humans, pigs, and both humans and pigs.

^b^ A total of 31 households had cases of either pig *T*. *solium* exposure-positive, human *T*. *solium* exposure-positive or *T*. *solium* taeniasis.

In three study districts, the prevalence of having a household latrine was relatively low ranging from 13% to 47%. This meant that outdoor defecation was a practice reported by between 15% and 74% of the study population. Our data showed that allowing pigs to free roam was common practice in Buon Bon, Krong Nang, and M’Drak. The percentage of pigs that consumed human feces was high in Buon Don and M’Drak (Table 2). Of 11 exposure-positive households for pigs, there were nine households in M’Drak. Pigs kept in seven of the nine households were allowed to roam freely within the village.

**Table 2:** Spatial characteristics of *T*. *solium* exposure and taeniasis. Pig management in the study districts of Dak Lak province.

Location	Total pigs	Free-roaming pigs		Human coprophagy	
		*n*	% (95% CI)	*n*	% (95% CI)
**Buon Don**	323	164	51 (45 to 56)	133	41 (36 to 47)
**Krong Nang**	447	31	6.9 (4.8 to 9.8)	14	3.1 (1.8 to 5.3)
**M’Drak**	511	211	41 (37 to 46)	193	38 (36 to 42)

### Distribution of pig *T*. *solium* exposure households

**Fig 3:**
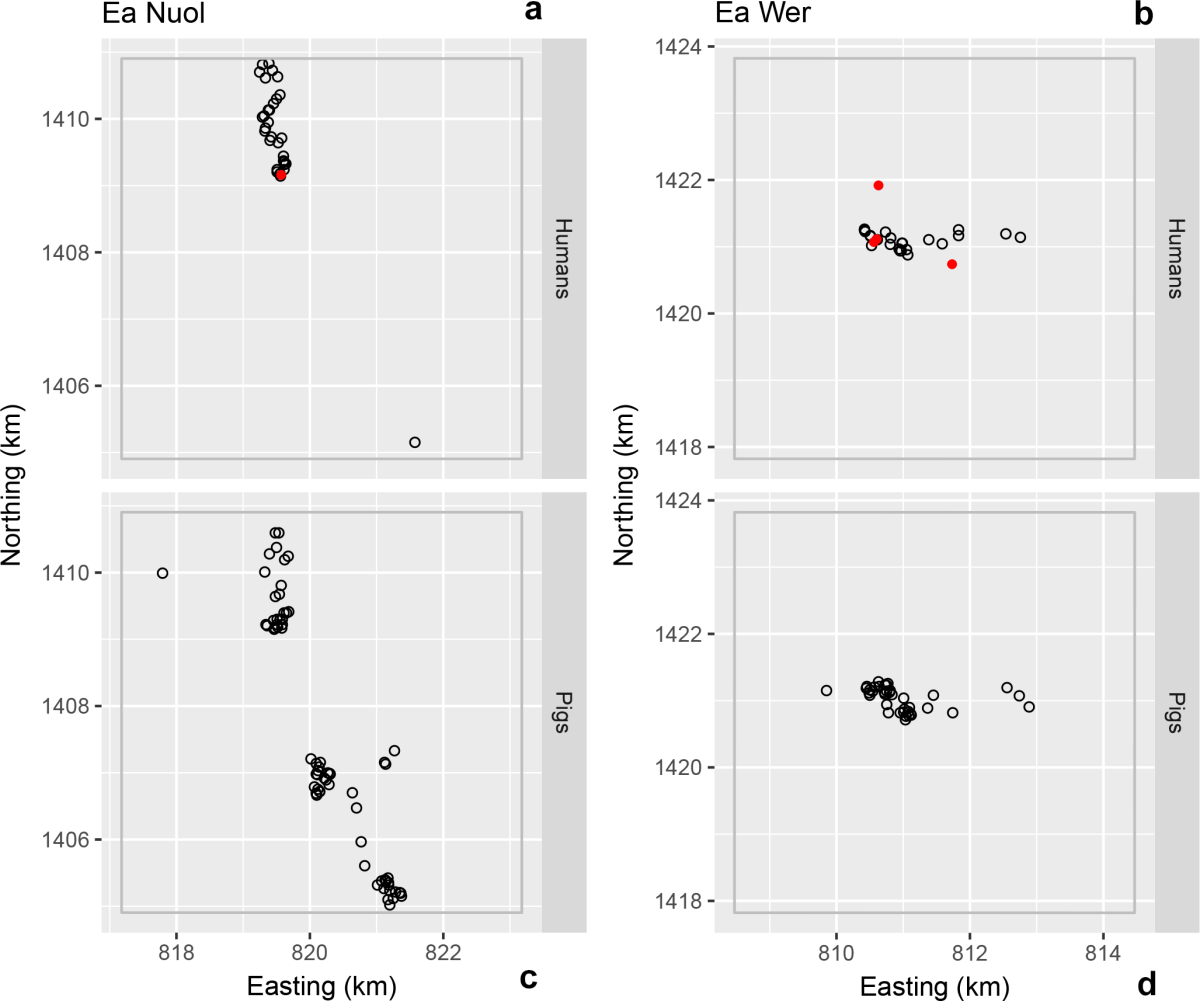
**Location of households for human (a and b) and pig (c and d) *T*. *solium* exposure in Ea Noul (a and c) and Ea Wer (b and d) villages in Buon Don district.** The solid red circles indicate the location of exposure-positive households; the open grey circles indicate the location of exposure-negative households.

**Fig 4:**
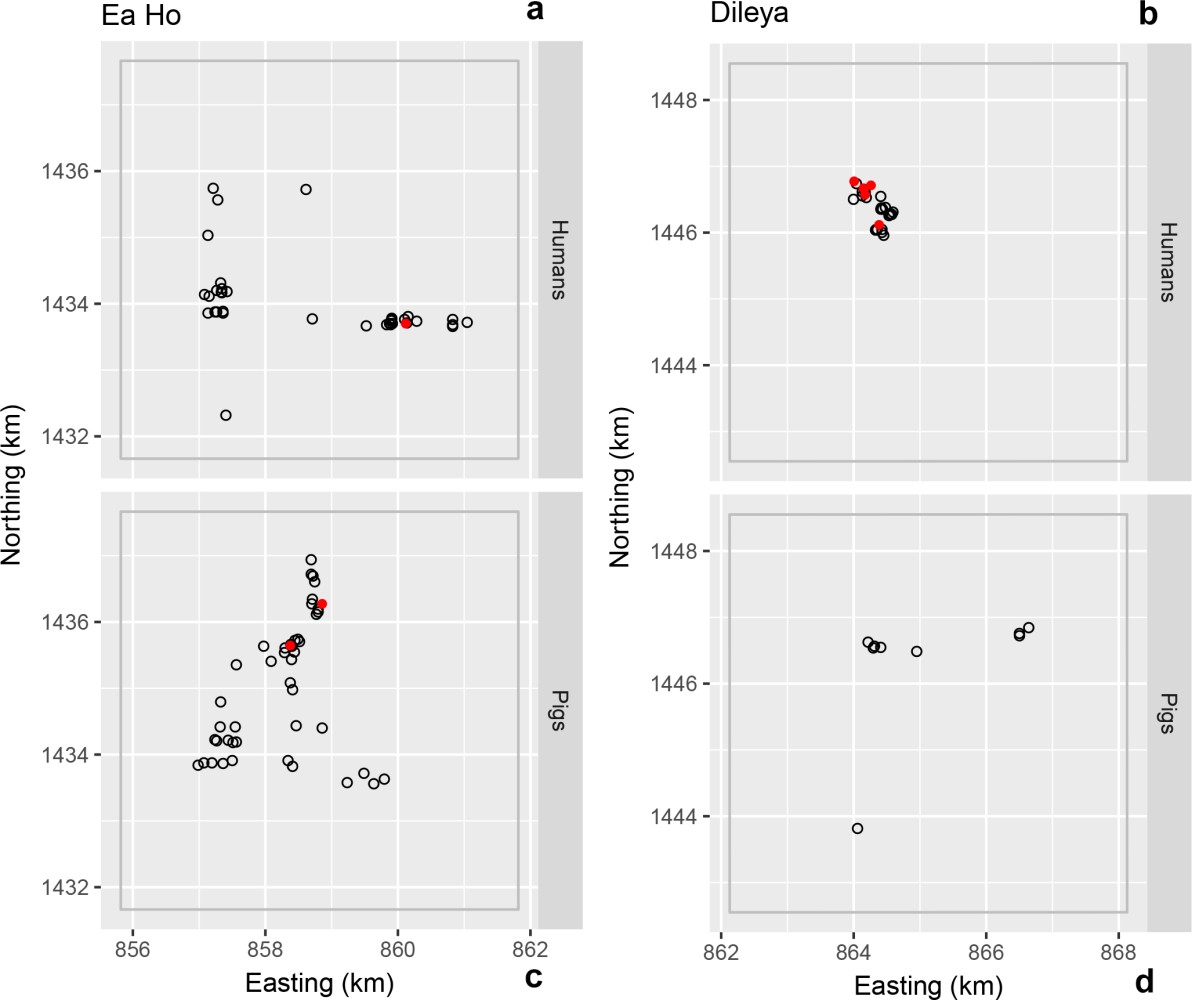
**Location of households for human (a and b) and pig (c and d) *T*. *solium* exposure in Ea Ho (a and c) and Dlieya (b and d) villages in Krong Nang district.** The solid red circles indicate the location of exposure-positive households; the open grey circles indicate the location of exposure-negative households.

**Fig 5:**
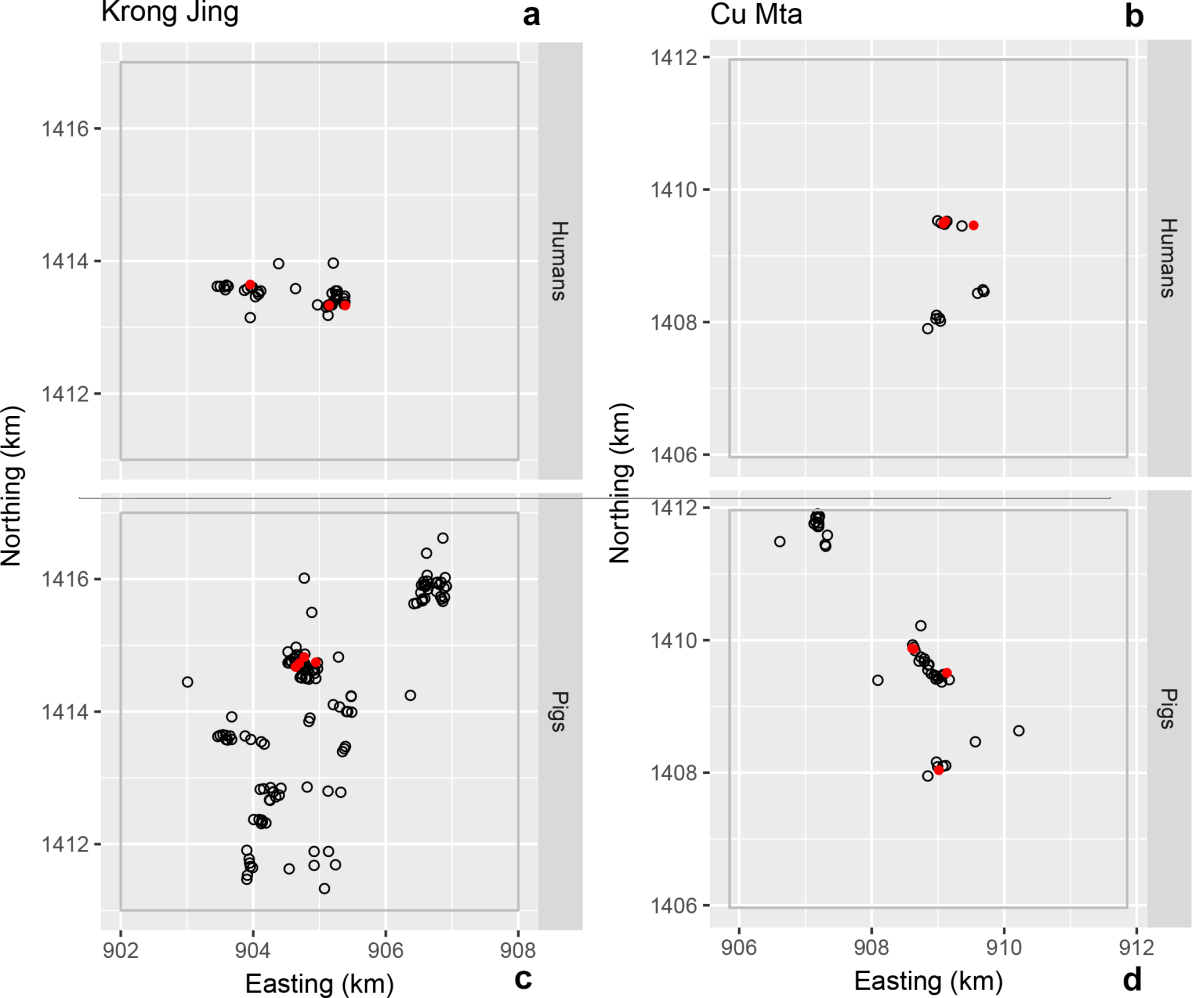
**Location of households for human (a and b) and pig (c and d) *T*. *solium* exposure in Krong Jing (a and c) and Cu Mta (b and d) villages in M’Drak district.** The solid red circles indicate the location of exposure-positive households; the open grey circles indicate the location of exposure-negative households.

**Fig 6:**
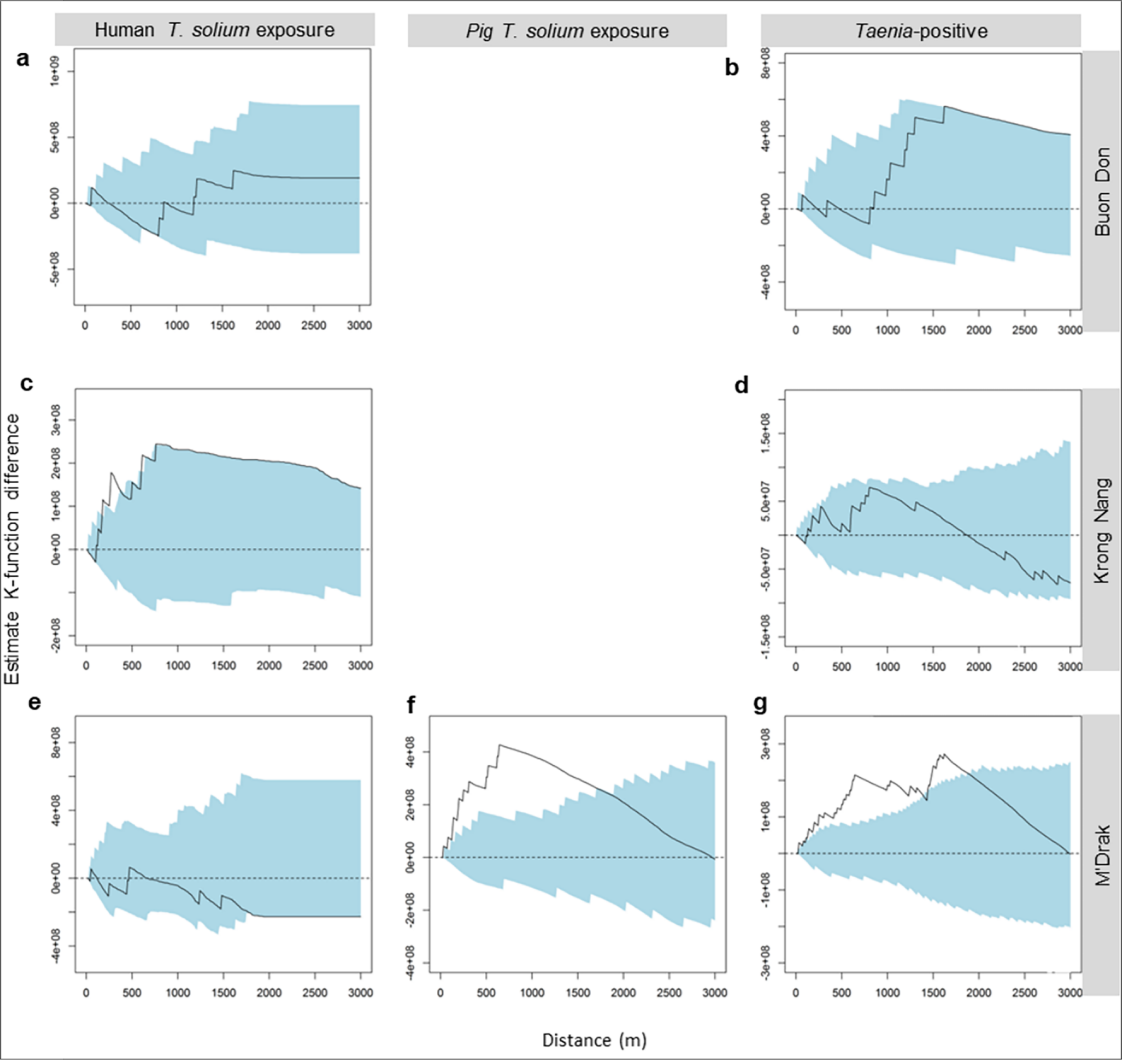
Estimated K-function differences for human and pig *T*. *solium* exposure- and *Taenia*-positive households in three studied districts. Horizontal dotted lines at *D*(*h*) = 0 provides a reference for *D*(*h*) under complete spatial randomness. The solid line indicates the observed value of *D*(*h*). The light blue areas indicate the 95% tolerance limits for *D*(*h*) estimated from 1000 Monte Carlo simulations. Panel (a), (c) and (e) showing the estimation for human *T*. *solium* exposure respectively in Buon Don, Krong Nang and M’Drak; panel (b), (d) and (g) for *Taenia*-positive respectively in Buon Don, Krong Nang and M’Drak and panel (f) for *T*. *solium* exposure in pigs in M’Drak.

No exposed pigs were detected in Buon Don (Fig 3C and 3D) and two exposed pigs were detected in Krong Nang (Fig 4C and 4D). In M’Drak, the four exposed pigs that were detected were within a distance of 250 m of each other (Fig 5C) and pairs of exposure-positive pig households were less than 100 m apart (Fig 5D). The K-function difference plot for *T*. *solium* exposure in pigs in M’Drak shows *K*(*h*)_*positive*_ in excess of *K*(*h*)_*negative*_ up to a distance of 1500 m (Fig 6F).

### Distribution of human *T*. *solium* exposure households

There were small numbers of human *T*. *solium* exposure-positive households in close proximity in Krong Nang (Fig 4B). The K-function difference plot for exposure to *T*. *solium* in humans in Krong Nang supported this observation, where *K*(*h*)_*positive*_ was in excess of *K*(*h*)_*negative*_ up to a distance of 200 to 300 m (Fig 6C). The spatial distribution of human exposure-positive households in Buon Don (Fig 3A and 3B) and M’Drak (Fig 5A and 5B) were more regularly distributed; there was no evidence of significant differences between *K*(*h*)_*positive*_ and *K*(*h*)_*negative*_ up to a distance of 3000 m (Fig 6A and 6E).

### Distribution of *Taenia*-positive households

**Fig 7:**
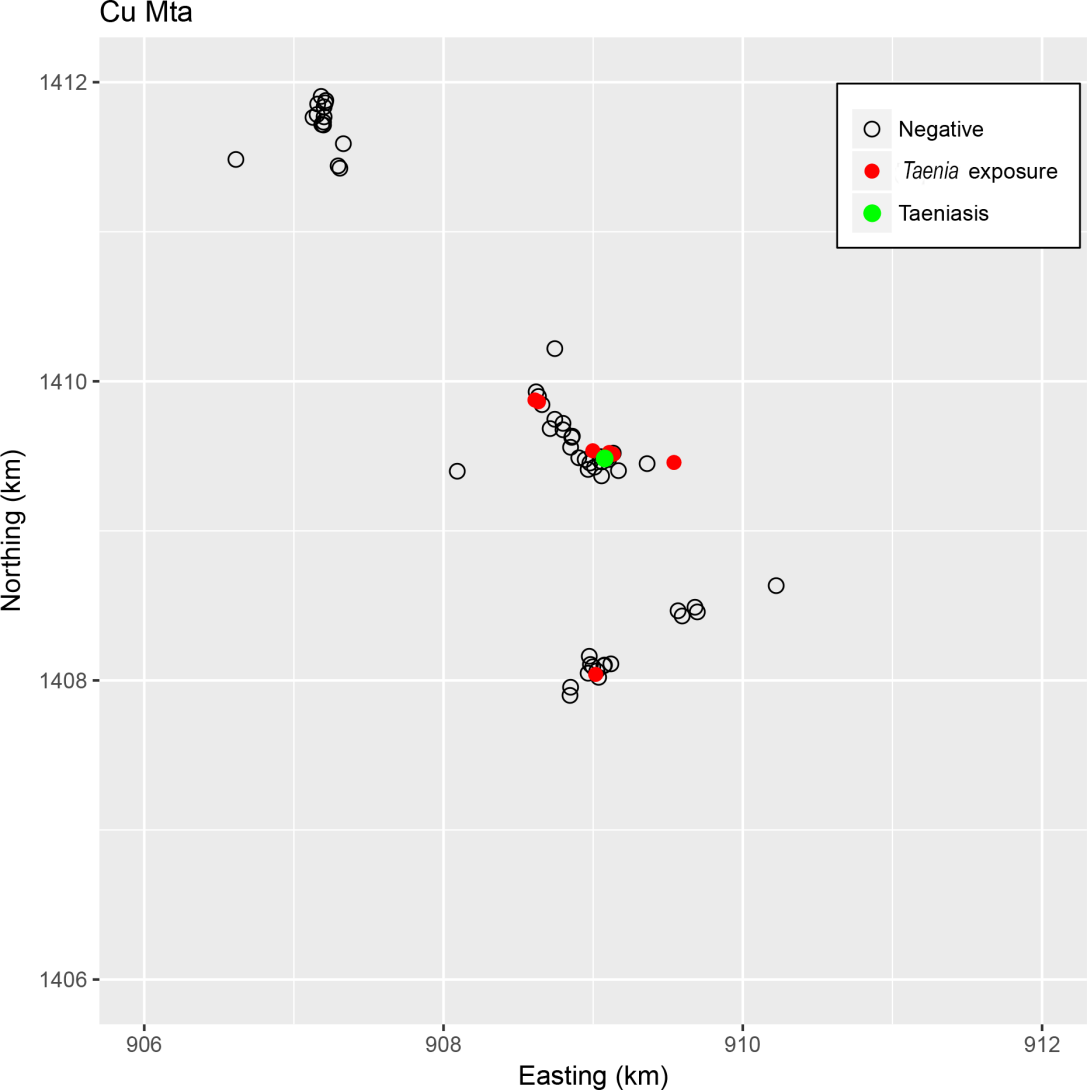
Location of *T*. *solium* taeniasis- and exposure-positive households. A group of cysticercosis-positive households (red circles) occurred in close proximity of a taeniasis-positive household (green circles) in the village of Cu Mta of M’Drak district. The taeniasis-positive household (green circles) had a case of cysticercosis.

When we considered households that were either human *T*. *solium* exposure, taeniasis or pig *T*. *solium* exposure as a single group, the K-function difference plot showed all *T*. *solium* exposure-positive and taeniasis-positive households were aggregated up to a distance of 1000 m in M’Drak (Fig 6g). Similar associations were evident in Buon Bon and Krong Nang but the observed K-function difference plot did not exceed the simulation envelope limits at any distance (Fig 6B and 6D). On inspection, however, we observed a group of taeniasis- and exposure-positive households in close proximity to each other in the village of Cu Mta in M’Drak district (Fig 7).

## Discussion

This study describes the fine scale spatial distribution of *T*. *solium* exposure in pigs and humans in Vietnam for the first time. The geographic distribution of *T*. *solium* exposure- and taeniasis-positive households varied markedly across the districts of Buon Don, Krong Nang and M’Drak of Dak Lak province. A prominent feature of this data is that the prevalence of *T*. *solium* exposure in both species and *T*. *solium* taeniasis was relatively low (in humans 9 exposure- and 2 taeniasis-positive households per 100 households at risk; in pigs 3 exposure-positive households per 100 households at risk) making it difficult to definitively identify characteristics of the spatial distribution of positive households that are likely to exist across all districts of Dak Lak, and indeed all districts of Vietnam.

Spatial aggregations of *T*. *solium* exposure-positive households for humans occurred in some (the village of Ea Wer in Buon Don district (Fig 3B), Dlieya in Krong Nang district (Fig 4B) and Krong Jing and Cu Mta in M’Drak (Fig 5A and 5B)), but not all, of the villages in the three study districts. Our K-function difference plots showed that *T*. *solium* exposure-positive households for humans showed the same pattern of spatial dependence as *T*. *solium* exposure-negative households in Buon Bon and M’Drak (Fig 6A and 6E). In Krong Nang, compared with human exposure-negative households, human exposure-positive households were aggregated up to a distance of 200 to 300 m (Fig 6C). We speculate that if the prevalence of exposure was higher in Buon Don and M’Drak and sufficient resources were available to allow larger sample sizes in each of the two districts to be collected, a similar pattern of spatial dependence would be evident.

Although spatial aggregation of *T*. *solium* exposure-positive households for humans in Krong Nang was beyond that expected by chance (and entirely due to a collection of five positive households in the village of Dlieya), its overall magnitude was relatively small (Fig 6C). Dlieya is small village comprised of less than 200 households in a remote area of Krong Nang. In Dlieya the number of individuals per household was considerably larger than that of the other villages in the study (median 5; minimum 2 to maximum 11) and a notable feature was that it was common for several generations of a family to live together in close proximity, and a highly prevalent custom was that food was shared with neighbours and relatives on a daily basis, often associated with community ceremonies (e.g. weddings and anniversary of deaths). In the district of Krong Nang, houses are typically surrounded by a large garden comprised of coffee or pepper trees. We hypothesize that in this district, where a high proportion of the study population were known to defaecate outdoors, individuals were more likely to defecate in their own garden (as opposed to communal areas) which means that it was more likely for *T*. *solium* eggs to be present in close spatial proximity to a given household where exposure-positive individuals were present. We speculate that the anthropological and fine-scale environmental characteristics of Dlieya were sufficient to allow spatial clustering of human exposure infection to be detected even in the presence of a modest sampling effort (*n* = 30 households).

Spatial aggregations of exposure to *T*. *solium* in pigs occurred but this was infrequent. In three study districts, there was a single aggregation of pig exposure-positive households in M’Drak (Fig 5C). Our K-function difference plot for M’Drak (Fig 6F) showed pig exposure-positive households were clustered within a distance of 1500 m. Presumably, this was due to the larger range over which free-roaming pigs forage. Copado et al., 2004 [26] reported that free-roaming pigs travel daily within a distance ranging from 1000 to 3000 m. In a 12 hour period, pigs traveled a distance of up to 4000 m and spent, on average, 47% of their time outside of their homestead [28]. Our findings are supported by those of Ngowi et al. (2010) [12] who conducted a cross-sectional study of cysticercosis in 784 pig-owning households in northern Tanzania. In the study of Ngowi et al. (2010) it was shown that porcine cysticercosis was clustered within the distance of 600 m and 10 km. Morales et al. (2008) [29] conducted a cross-sectional study of 562 pigs in the state of Morelos in Mexico in 2003. In this study the prevalence of porcine cysticercosis was relatively high (13%; 95% CI 11 to 17) and while free-roaming pigs had a greater risk of being cysticercosis-positive, no geographical clustering of positivity was found.

Spatial clustering of *T*. *solium* exposure in pigs in M’Drak could have been associated with the age of the resident pig population, the absence of pigsties and the regular habit of coprophagy amongst pigs. In total, there were 11 *T*. *solium* pig exposure-positive households in Dak Lak. Nine of these 11 positive households were in M’Drak, aggregated in groups of two to four households (Fig 5C and 5D). Of the nine *T*. *solium* pig exposure-positive households in M’Drak, in seven households pigs were allowed to roam freely within the village, increasing the chance of exposure to *T*. *solium* eggs. The terrain in M’Drak is generally flat. The quality of the soil is poor supporting predominantly natural grasslands. For these reasons, the practice of allowing pigs to free roam is more common compared with the two other districts. M’Drak had a high proportion of pigs that were not confined (17% [211 of 1281], 95% CI 15 to 19). Of the total number of pigs sampled in this study, 511 (40%) were from M’Drak. Of the 511 M’Drak pigs that were sampled, it was reported that 193 (38%) consumed human feces and 211 (41%) regularly scavenged for food (Table 2).

When we considered households that were human and/or pig *T*. *solium* exposure-positive or taeniasis positive as a single group, our K-function difference plot showed these positive households were aggregated up to a distance of 1000 m in M’Drak (Fig 6G), but not in Buon Bon (Fig 6B) and Krong Nang (Fig 6D). O’Nea at al. (2012) and Pray et al. (2017) showed that human and/or porcine cysticercosis cases were strongly associated with the presence of tapeworm carriers [14,15]. Individuals and pigs living in close proximity to tapeworm carriers are more likely to be infected with *T*. *solium* cysticercosis [11,15,30]. Given this unique data set, with contemporary sampling of humans and pigs, it was of interest to us to determine if there was a spatial dependence between *T*. *solium* exposure- and *T*. *solium* taeniasis-positive households. Although households that had either *T*. *solium* exposure- or taeniasis-positive cases were spatially aggregated, we were unable to identify an association between the two because of the extremely low number (*n* = 4) of taeniasis-positive households across the three study districts. On inspection we observed a group of taeniasis- and exposure-positive households in close proximity to each other in the village of Cu Mta in M’Drak district (Fig 7). Madinga et al. (2017) [26] and Morales et al. (2008) [29] indicated that there was no spatial correlation of *T*. *solium* exposure in pigs and *T*. *solium* taeniasis.

Spatial aggregations of human and pig exposure-positive households occurred in some, but not all, of the villages in the three study districts. We can only speculate about the reasons for this pattern, as discussed above. Since cysticercosis occurs throughout Vietnam [17] it is likely that *foci* of infection are present in other areas. The relatively low prevalence of exposure to *T*. *solium* indicates that massive deworming programs in the communities of Dak Lak province are, for the most part, unnecessary. Instead, we recommend that if a human is identified as *T*. *solium* positive then either: (a) individuals resident in the immediate area should be tested to rule out the presence of an exposure or infection cluster; or (b) anthelmintic treatment is offered to individuals resident within a 2000 m radius of the identified case. With respect to the second approach, privacy issues would need to be handled appropriately, particularly in small communities.

A limitation of this study was that for logistic reasons sampling of humans and pigs were carried out independently resulting in a lack of overlap of the locations of households where humans and pigs were sampled (see, for example, Ea Nuol in the district of Buon Don, Fig 3A and 3C). While this limited our ability to identify an association (if any) between human and pig *T*. *solium* exposure-positive households, assessment of the spatial dependence of exposure status by species (human, pigs) was possible. Although households that had either *T*. *solium* exposure- or taeniasis-positive cases were spatially aggregated, we were unable to quantify their spatial association due to the extremely low number of *T*. *solium* taeniasis-positive households.

## Supporting information

S1 Checklist STROBE checklist. (DOC)
